# Molecular survey of certain protozoan agents that cause diarrhea in children in Sudan

**DOI:** 10.12688/f1000research.123652.1

**Published:** 2022-11-29

**Authors:** Mosab Adam, Hongwei Shen, Khalid-A Enan, Hao Wang, Azza B. Musa Musa, Abdel R. El Hussein, Isam M. Khidir, Xuejun Ma

**Affiliations:** 1 National Institute for Viral Disease Control and Prevention, Chinese Center for Disease Control and Prevention, China., beijing, China; 2 Department of Virology, Ministry of Higher Education and Scientific Research, Khartoum, Sudan,, khartoum, Sudan; 3 Futian District Center for Disease Control and Prevention, Shenzhen, China., Shenzhen, China; 4 Department of Microbiology and Parasitology,, Faculty of Medicine, University of Khartoum, Khartoum, Sudan, khartoum, Sudan

**Keywords:** Diarrhea, Detection, Parasitic, Protozoan, Pathogens, Childhood

## Abstract

Introduction

Diarrhea is a significant health problem in third world countries; identification of causative agents of diarrhea is essential to apply measures to prevent and control this disease. In addition, scant data are available regarding childhood diarrhea in Sudan. Our research aimed to determine the incidence of specific protozoan pathogens ( *Entameobia histolytica*, *Cryptosporidium* spp., and *Gardia lambelia*) among the young (aged less than five years) in Khartoum, Sudan.

Methods

We conducted a parasitological cross-sectional survey, and stool samples from 437 patients were examined for *E. histolytica, C.  parvum, and G. lambelia* using a multiplex real-time PCR method.

Results

Of the 437 stool samples tested, infection with intestinal parasite was found in 155 (35.5%) cases, and co-infection was identified in 16 (3.7%) cases. *G. lambelia* (18.8%) and *C. parvum* (15.8 %) were the most frequently identified parasites, followed by *E. histolytica* (0.5%). The highest and lowest rates of parasitic infections were seen in the less than two years age group (32.7%), and in the 2–4-year-old group (2.7%), the male children showed higher rates of infections (23.7%) compared to females (11.7%). The incidence of protozoan infection was higher (37.7%) in the rainy season (August to December) (32.7%) in contrast with that( 2.7%) in the dry season (April to June) .

Discussion

Our present study demonstrated the high prevalence of *G. lambelia* and *C. parvum* in children with diarrhea in Khartoum State and the multiplex real-time technique's usefulness in disclosing pathogenic protozoal agents. Our result highlighted the necessity of developing intervention measurement and control strategies to deal with childhood parasitic diarrhea in this region.

## Introduction

Diarrhea is defined as passing soft, loose, or watery feces three times or more in 24 hours; it is usually a result of the consumption of pathogen-contaminated water or food. ^1^^,^^2^ Diarrhea remains a major cause of mortality and morbidity in children in third-world countries. ^1^^,^^3^^–^^5^ More than 1 billion episodes of diarrhea occur annually, resulting in approximately 2.5 million deaths in children aged less than 5 years in developing countries. ^1^^,^^2^^,^^5^^,^^6^ Where diarrhea is considered the third most common cause for young children to visit health centers, some of the underlying conditions found in the community of most developing countries, including malnutrition and poor hygiene, may increase the risk of experiencing diarrheal disease. ^2^ In developed countries, the availability of modern technologies and suitable water supply has led to a decline in global death due to diarrhea; however, despite the substantial effort to supply modern technology and management practices, diarrhea in Africa is still unacceptably ranked as the second cause of death among young children. ^1^^,^^7^^–^^9^ Despite the high morbidity of childhood diarrhea in Sudan, the knowledge of the parasitic causative agents is scant. Parasitic protozoans that infect the intestinal tract in developing countries include *Cryptosporidium* spp., *G. lambelia*, and *E. histolytica* the agents that cause cryptospordiasis, giardiasis, and amoebiasis respectively, which are considered prime for diarrheal diseases in children under 5 years old. ^15^ The limited specificity and sensitivity of the microscopic method commonly used in most laboratories in Sudan decreased the detection rate of parasitic infections. As a result, there is little information about the precise incidence of diarrhea and causative protozoan agents.

This study aimed to explore the incidence of some protozoan organisms ( *Cryptosordium parvum*, *Giardia lambelia*, *Giardia lambelia*, and *Entameobia histolytica*) that produce acute diarrheal illness among young children using molecular techniques.

## Methods

This cross-sectional study was co-conducted at the Central Laboratory, Ministry of Higher Education and Research, Sudan, and the National Institute for Viral Disease Control and Prevention, China Center for Disease Control and Prevention, China (CDC), Beijing, China. A total of 437 fecal samples (one per patient) comprising 276 boys and 161 girls, who mostly live in a rural area, were collected in a dry, clean plastic container during two different seasons (the hot, dry season from April to June, and the rainy season from August to December) in the year 2014 at Khartoum teaching hospitals. The stool specimens were kept at −20°C until tested at the beginning of 2015. The frozen samples were then transported on dry ice to the China CDC, Beijing, China. Children admitted to hospitals had been clinically diagnosed with acute diarrhea ranging from 1 to 4 days before the sample collection. The participants were aged ≤2 years (403, 92.2%); >2–≤4 years (32, 7.3%), and >4–˂5 years (2, 0.5%). Patient data were collected through a structured questionnaire, including age, gender, and season. The study was approved by the ethical committee of the Sudan Academy of Sciences (Approval number (2367) and informed written permission was obtained from the parents or guardians of the enrolled children.

### Nucleic acid extraction

Parasite DNA was extracted from 200 μL of 10% fecal suspension prepared in phosphate buffer saline using QIAamp ^®^ Fast DNA Stool Mini Kit (Qiagen, Hilden, Germany) according to the manufacturer’s instructions. The extracts were eluted in 60 μL of DNase-free water, immediately aliquoted in 20 μL, and kept at −80°C.

### PCR amplification and parasite detection

**Primers and probes of multiplex real-time PCR**

A total of three primer pairs and three probes for the simultaneous detection of *E. histolytica*, *C. parvum*, and *G. lamblia* were used. ^10^ Table 1 shows the oligonucleotides sequence of the primers, probes and target genes, and amplicon sizes.

**Table 1.** The nucleotide primers and probes for multiplex real-time PCR used in this study

Organism	Target gene ^a^	Primer sequence (5′-3′) ^b^	Probe (5′-3′)
*E. histolytica* ^5^	SSU rRNA	F: ATTGTCGTGGCATCCTAACTCA R: GCGGACGGCTCATTATAACA	VIC-TCATTGAATGAATTGGCCATTT ^19^
*G. lamblia* ^5^	SSU rRNA	F: GACGGCTCAGGACAACGGTT R: TTGCCAGCGGTGTCCG	FAM-CCCGCGGCGGTCCCTGCTAG ^19^
*C. parvum* ^5^	DNA-like	F: CGCTTCTCTAGCCTTTCATGA R: CTTCACGTGTGTTTGCCAAT	Texas Red-CCAATCACAGAATCA ^19^ TCAGAATCGACTGGTATC ^16^

^a^ SSU rRNA, small subunit ribosomal RNA.

^b^ F, forward; R, revers.

**Multiplex real-time PCR**

Real-time PCR was performed with a Multiplex PCR kit (Qiagen, Hilden, Germany) in a 20 μL volume containing 6.25 pmol of each *E. histolytica-*specific primers, 6.25 pmol of each *G. lamblia*-specific primers, 25 pmol of each *C. parvum*-specific primers, 1.75 pmol of *E. histolytica*-specific VIC-TaqMan probe, 2.5 pmol of *G. lamblia*-specific FAM-TaqMan probe, 8.75 pmol of *C. parvum*-specific Texas Red-TaqMan probe. Amplification consisted of 15 min at 95°C, 40 cycles of 15 s at 95°C, 30 s at 60°C, and 30 s at 72°C. The iCycler real-time detection system (Bio-Rad) performed amplification, detection, and data analysis.

## Result

**Figure 1.**
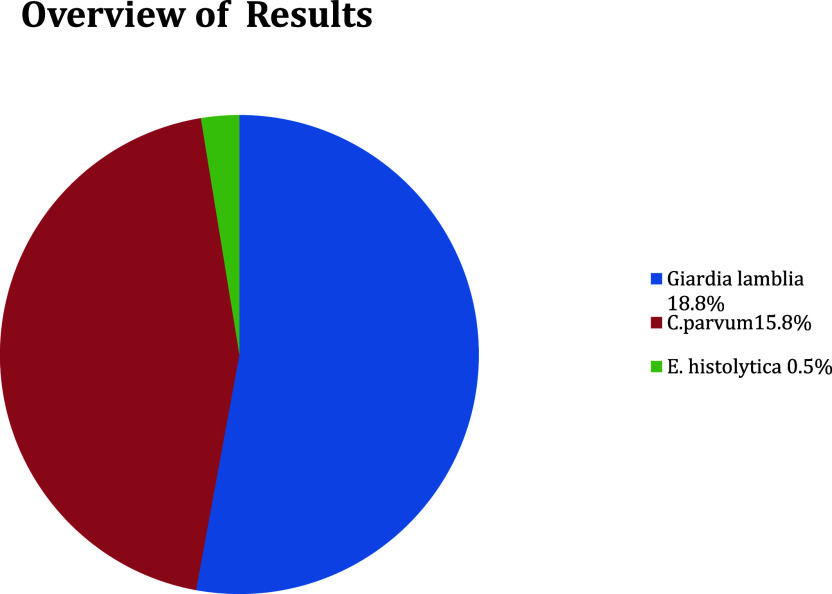
Overview of protozoal parasite infections.

A potential protozoal parasite was diagnosed in 155/437 (35.5%) cases, among which the highest prevalence was *G. lamblia* (82/437, 18.8%), followed by *Cryptosporidium* spp. (69/437, 15.8%), and *E. histolytica* (4/437, 0.5%) ( Figure 1). The highest rate of parasitic infection was seen in the ≤2 years group (32.7%) and much lower in the >2–≤4 years old group (2.7%) ( Table 2). In contrast, the protozoal parasite was not detected in the age group of >4–˂5 years. Among children with parasitic infections, 23.7% were male, while 11.7% were female ( Table 3). As much more samples were collected from boys (276) than from girls (161) in the age group ≤2 years (403, 92.2%), the comparisons between these variables were not relevant. The incidence of protozoan parasitic infection was higher in the rainy season (August to December) than in the dry season (April to June) (32.7% and 2.7%, respectively) ( Table 4). 

**Table 2.** Frequency of protozoan pathogens in children with diarrhea in Khartoum among the age.

	Age in years			P-value
	0-2	2-4	4-5	
***G. lambelia***	17.2%	1.6%	0%	0.715
***Cryptosporidium* spp.**	14.6%	1.1%	0%	0.828
***E. histolytica***	0.9%	0%	0%	0.843
**Total**	32.7%	2.7%	0%	

**Table 3.** Frequency of protozoan pathogens in children with diarrhea in Khartoum among the gender.

	Gender		P-value
	M	F	
***G. lambelia***	14.2%	4.6%	0.010
***Cryptosporidium* spp.**	9.4%	6.4%	0.483
***E. histolytica***	0.2%	0.7%	0.112
**Total**	23.8%	11.7%	

**Table 4.** Frequency of protozoan pathogens in children with diarrhea in Khartoum among the Seasons.

	Season		P-value
	Autumn	Summer	
***G. lambelia***	16.9%	1.8%	0.391
***Cryptosporidium* spp.**	14.9%	0.9%	0.064
***E. histolytica***	0.9%	0%	0.446
**Total**	32.7%	2.7%	

Infection with one type of parasite was found in 139 cases (31.8%), while parasite co-infection was detected in 16 patients (3.7%), which involved *E. histolytica* and *G. lambelia* in two cases, and *G. lamblia* and *Cryptosporidium* spp. in 14 cases ( Table 5).

**Table 5.** Frequency of samples with co-infections.

Pathogen	No. of co-infections (%)
*G. lamblia* and *C. parvum*	14 (3.2%)
*E. histolytica* and *G. lambelia*	2 (0.5%)
Total	16 (3.7%)

## Discussion

Gastrointestinal protozoan parasites are still posing common health problems, mainly in children aged less than 5 years worldwide. The rapid and accurate identification of protozoan parasites is a big challenge in many developing countries. The real-time multiplex PCR technique that provides concurrent detection of all protozoal parasites used herein was an exceedingly powerful laboratory system, enabling rapid, sensitive, precise, and inexpensive parasite detection.

This study was conducted during two seasons (autumn and summer), from April to December 2014, in Khartoum State, Sudan. The present study aimed to determine the prevalence of certain protozoan parasites linked with acute gastroenteritis in stool samples from children less than 5 years old using a multiplex real-time PCR assay developed in a previous study. ^10^

Among the 437 fecal specimens examined, 276 were collected from male children and 161 from females, amounting to a male-to-female ratio of 1:1.7. Most of the samples were from less than ≤2 years old (403, 92.2%), followed by >2–≤4 years (32, 7.3%) and >4–˂5 years (2, 0.5%).

As they showed in Table 2, the gender distribution among the *G. lambelia*-positive samples was 14.2% in males and 4.6 % in females (P<0.01), indicating a statistically significant difference among the gender group that supports the statement of Khwam H. ^11^

The most significant number of samples were from the age group ≤2 years (403, 92.2%), followed by >2–≤4 years (32, 7.3%) and >4–˂5 years (2, 0.5%). The result of our study indicates the highest prevalence of protozoal diarrhea (32.7%) was detected in the age group of ≤2 years flowed by >2–≤4 years (2.7%), and no protozoan pathogen was found in the age group of >4–˂5 years; however, these results could be explained by the fact that most of our samples were collected from the age group ≤2 years in which the decline of the maternal immunity with an age risk factor of diarrhea infection. ^4^^,^^12^ The highest number of positivity was detected in the samples of boys less than two years old. The reason for the bias in the numbers of children with diarrhea (boys of ≤2 years) admitted to hospitals is not clear and needs to be further investigated to determine whether or not it is the pattern of childhood diarrhea in Sudan. The contaminated hands and bad hygiene may contribute to the transmission of food borne infection in these children, which was in agreement with the investigation in Nepal, where the highest prevalence of parasitic diarrhea was found in the age group of fewer than two years. ^13^ But our result differed from another study in Khartoum ^13^ in which the major group of infections was in >4–˂5 years old children, and this may again be due to statistical bias because most of our samples were collected from the age group of ≤2 years; This should be avoided in future studies by equalizing the age group of the study or by using a larger sample size.

The result revealed a higher prevalence (35.5%) of protozoan diarrhea disease compared with other studies conducted in Khartoum state (16%). ^14^ and in other developing countries, including Nepal and Ethiopia (0.7%, 15.6%, respectively). ^13^^,^^15^ In comparison, the incidence was lower than that in Tanzania and South Africa (55.6%, 68%, respectively) ^5^^,^^16^ and close to that reported in the Gaza strip (39%). ^17^ To our knowledge, our study is the first to demonstrate a high prevalence of *Cryptosporidium* (15.8%) in Sudan. The diagnosis of *Cryptosporidium* used to depend on the Ziehl−Neelsen stain, and it was neglected mainly by our laboratories until we used a sensitive molecular assay that increased the detection rate of these agents.

The most prevalent protozoan detected in the present investigation was *G. lamblia*, with a prevalence of 18.8%, which is higher than in the study conducted in Khartoum State (15.8%). ^14^ Its prevalence was followed by *Cryptosporidium* spp. (15.8%) and *E. histolytica* (0.9%). This result was consistent with previous findings in developing countries, including India, Gaza and Nigeria ^4^^,^^17^^,^^18^ Infection with a single protozoan parasite was found in 139 cases (31.8%) cases; co-infection was found in 16 cases (3.7%). The study of co-infection on clinical severity was not studied in these patients. However, it has been reported that no significant variation was reported in the clinical symptoms of patients with co-infections compared with those with single infections. ^19^ Our study showed that the incidence of a protozoan parasite is higher in autumn (wet) than that in summer (dry), which was in accordance with the study conducted in Khartoum state. ^14^ It should be noted that no protozoan pathogen was detected in 282 stool samples (64.6%) which were likely due to infections with other pathogens like viruses and bacteria and also may be due to noninfectious reasons like hypersensitivity to certain food ingredients and weaning diarrhea that result of the inability of an underdeveloped child intestine to metabolize the food. Poor hygiene and sanitation and lack of proper toilets may facilitate these infections.

The present study furnished some crucial insights into the protozoan cause of child diarrhea in Khartoum State. Findings of this study will certainly help us understand the importance of parasitic diarrhea in younger than five years of children, and these findings are valuable in developing measures to improve the health condition of the young children. Furthermore, this study calls for the establishment of sensitive and specific molecular techniques, such as multiplex PCR, for the detection of the protozoan pathogen in a clinical setting.

## Authors contributions

Mosab, Isam, and Xuejun designed the experiment; Mosab and Hong-do the lab experiment; Azza analyzed the data; Mosab, Khalid, and Abdel collected the samples. Mosab Hong and Abdel wrote the article.

## Data Availability

Figshare: Underlying data for “Molecular survey of certain protozoan agents that cause diarrhea in children in Sudan”, https://doi.org/10.6084/m9.figshare.21201676.v3. ^20^ This project contains the following underlying data:
•
Data information.xlsx Data are available under the terms of the Creative Commons Attribution 4.0 International license (CC-BY 4.0).
