# Molecular survey of certain protozoan agents that cause diarrhea in children in Sudan

**DOI:** 10.12688/f1000research.123652.2

**Published:** 2024-02-28

**Authors:** Mosab Adam, Hongwei Shen, Khalid-A Enan, Hao Wang, Azza B. Musa Musa, Abdel R. El Hussein, Isam M. Khidir, Xuejun Ma

**Affiliations:** 1 Department of Virology, Ministry of Higher Education and Scientific Research, Khartoum, Sudan,, khartoum, Sudan; 2 National Institute for Viral Disease Control and Prevention, Chinese Center for Disease Control and Prevention, China., beijing, China; 3 Futian District Center for Disease Control and Prevention, Shenzhen, China., Shenzhen, China; 4 Department of Microbiology and Parasitology,, Faculty of Medicine, University of Khartoum, Khartoum, Sudan, khartoum, Sudan

**Keywords:** Diarrhea, Detection, Parasitic, Protozoan, Pathogens, Childhood

## Abstract

**Introduction:**

Diarrhea is a significant health problem in Third World. Identification the pathogen that cause diarrhea is vital for measures to prevent and control this disease. There are also very few reports of diarrhea in Sudan. Our study aimed to determine the Prevalenceof specific protozoan pathogens ( *Entameobia histolytica*, *Cryptosporidium* parvum., and *Giardia lamblia*) in children in Khartoum, Sudan.

**Methods:**

We conducted a cross-sectional survey among children under five years of age hospitalized with acute diarrhea between April and December 2014. Diarrheastool samples were collected and examined *E. histolytica, C. parvum, and G. lamblia* using a multiplex real-time PCR.

**Results:**

437 acute children were included in this study; the higher Prevalence of diarrhea was in the age less than ≤2years old (403,92.2%). The male-to -female ratio in this study was 1:1.7. infection with intestinal parasite was found in 155 (35.5%) cases, and co-infection was detected in 16 (3.7%) cases. *G. lamblia* (18.8%) and *C. parvum* (15.8 %) were the most frequently identified parasites, followed by *E. histolytica* (0.9). The parasite infection rate was highest and lowest in the under 2-year-old group (32.7%), and in the 2–4-year-old group (2.7%),the infection rate was higher was higher in boys (23.7%) than in girls (11.7%). tThe incidence of protozoan infection was higher (37.7%) in the rainy season (August to December) (32.7%), corresponding with that in the dry Season (April to June). (2.7%)

**Discussion:**

Our present study demonstrated the high prevalence of *G. lamblia* and *C. parvum* in children with diarrhea in Khartoum region and usefulness of the multiplex real-time method in disclosing pathogenic protozoal agents. Our result highlighted the necessity of developing intervention measurement and control strategies to deal with childhood parasitic diarrhea in this region.

## Introduction

Diarrhea is defined as passing soft, loose, or watery feces three times or more in 24 hours; it is usually a result of the consumption of pathogen-contaminated water or food. ^1^ Diarrhea remains the leading cause of death and illness in children in third-world countries. ^2^^–^^4^ Around 1.7 billion cases of childhood diarrhea are reported each year, and diarrhea is estimated to have killed 500,667 children under the age of 5 worldwide in 2019. ^1^^,^^5^ Where diarrhea is considered the third most common cause for young children to visit health centers, some of the underlying conditions found in the community of most developing countries, including malnutrition and poor hygiene, may increase the risk of experiencing diarrheal disease. ^6^ In developed countries, the availability of modern technologies and suitable water supply has led to a decline in global death due to diarrhea; however, despite the substantial effort to supply modern technology and management practices, diarrhea in Africa is still unacceptably ranked as the second cause of death among young children. ^7^^–^^10^ Despite the high morbidity of childhood diarrhea in Sudan, the knowledge of the parasitic causative agents is scant. Parasitic protozoans that infect the intestinal tract in developing countries include *Cryptosporidium* spp., *G. lamblia*, and *E. histolytica*, respectively, the agents that cause cryptospordiasis, giardiasis, and amoebiasis, which are considered prime for diarrheal diseases in children under 5 years old. ^11^ The limited specificity and sensitivity of the microscopic method commonly used in most laboratories in Sudan decreased the detection rate of parasitic infections. As a result, there is little information about the precise incidence of diarrhea and causative protozoan agents.

This study aimed to explore the incidence of some protozoan organisms ( *Cryptosordium parvum*, *Giardia lamblia*, and *Entameobia histolytica*) that produce acute diarrheal illness among young children using molecular techniques.

## Methods

### Ethical considerations

The study was approved by the ethical committee of the Sudan Academy of Sciences (Approval number (2367) and written permission was obtained from the registered child’s parents or guardian.

### Design, area, and period of study

This cross-sectional study was co-conducted at the Central Laboratory, Ministry of Higher Education and Research, Sudan, and the National Institute for Viral Disease Control and Prevention, China Center for Disease Control and Prevention, China (CDC), Beijing, China during two different Seasons (the hot, dry Season from April to June (Summer), and the rainy Season from August to December (Autumn) in the year 2014 at Khartoum teaching hospitals.

### Participants (inclusion and exclusion criteria), sample collection and storage

A total of 437 fecal samples (one per patient) the children admitted to hospitals had been clinically diagnosed with acute diarrhea ranging from 1 to 4 days before the sample collection, aged less than five years, mostly lived in a rural area, the sample collected in a dry, clean plastic container. The stool specimens were kept at −20°C till tested in early 2015; frozen samples were sent via dry ice to the Center for Disease Control and prevention in Beijing, China.

### Data collection

Patient data including age, biological gender, and Season, were collected through a structured questionnaire.

### Nucleic acid extraction

According to the manufacturer’s instructions, parasite DNA was extracted from 200 μL of 10% fecal suspension prepared in phosphate buffer saline using QIAamp ^®^ Fast DNA Stool Mini Kit (Qiagen, Hilden, Germany). The extracts were eluted in 60 μL of DNase-free water, immediately aliquoted in 20 μL, and kept at −80°C.

### PCR amplification and parasite detection

**Primers and probes of multiplex real-time PCR**

Three primer pairs and three probes for the simultaneous detection of *E. histolytica*, *C. parvum*, and *G. lamblia* were used. ^11^ Table 1 shows the oligonucleotides sequence of the primers, probes and target genes.

**Table 1.** The nucleotide primers and probes for multiplex real-time PCR used in this study.

Organism	Target gene ^a^	Primer sequence (5′-3′) ^b^	Probe (5′-3′)
*E. histolytica*	SSU rRNA	F: ATTGTCGTGGCATCCTAACTCA R: GCGGACGGCTCATTATAACA	VIC-TCATTGAATGAATTGGCCATTT ^11^
*G. lamblia*	SSU rRNA	F: GACGGCTCAGGACAACGGTT R: TTGCCAGCGGTGTCCG	FAM-CCCGCGGCGGTCCCTGCTAG ^11^
*C. parvum*	DNA-like	F: CGCTTCTCTAGCCTTTCATGA R: CTTCACGTGTGTTTGCCAAT	Texas Red-CCAATCACAGAATCA ^11^ TCAGAATCGACTGGTATC ^11^

^a^ SSU rRNA, small subunit ribosomal RNA.

^b^ F, forward; R, revers.

**Multiplex real-time PCR**

Real-time PCR was performed with a Multiplex PCR kit (Qiagen, Hilden, Germany) in a 20 μL volume containing 6.25 pmol of each *E. histolytica-*specific primers, 6.25 pmol of each *G. lamblia*-specific primers, 25 pmol of each *C. parvum*-specific primers, 1.75 pmol of *E. histolytica*-specific VIC-TaqMan probe, 2.5 pmol of *G. lamblia*-specific FAM-TaqMan probe, 8.75 pmol of *C. parvum*-specific Texas Red-TaqMan probe. Amplification consisted of 15 min at 95°C, 40 cycles of 15 s at 95°C, 30 s at 60°C, and 30 s at 72°C. The iCycler real-time detection system (Bio-Rad) performed amplification, detection, and data analysis.

### Statistical analysis

The Chi-square test assessed differences in proportions. P values <0.05 were considered statistically significant.

## Result

### Demographic data on the population

A total of 437 children admitted to hospitals had been clinically diagnosed with acute diarrhea, Comprising 276 boys and 161 girls. The participants were aged ≤2 years (403, 92.2%), >2–≤4 years (32, 7.3%), and >4–˂5 years (2, 0.5%).

### Prevalence of parasitic infections

**Diagram 1.**
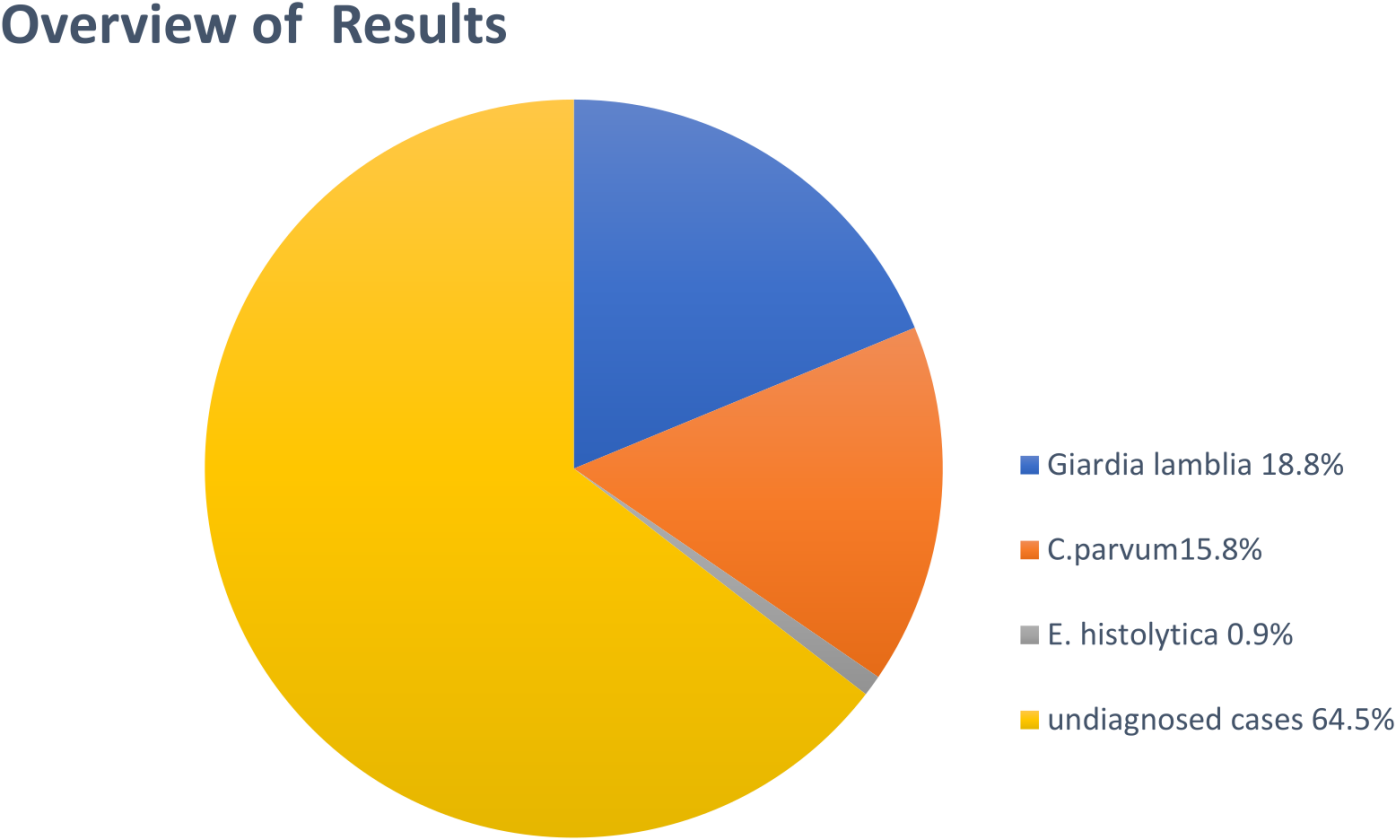
Overview of protozoal parasite infections.

A potential protozoal parasite was diagnosed in 155/437 (35.5%) cases, among which the highest prevalence was *G. lamblia* (82/437, 18.8%), followed by *C. parvum* (69/437, 15.8%), and *E. histolytica* (4/437, 0.9%) No protozoan pathogen was detected in 282 (64.5%) diarrheal cases ( Diagram 1).

### Distribution of parasitic infections by age, sex and seasonality

The highest rate of parasitic infection was seen in the ≤2 years group (32.7%) and much lower in the >2–≤4 years old group (2.7%) ( Table 2). In contrast, the protozoal parasite was not detected in the age group of >4–˂5 years. Among children with parasitic infections, 23.7% were male, while 11.7% were female ( Table 3). Diarrhea remains the leading cause and illness in children ≤2 years (403, 92.2%), the comparisons between these variables were not relevant. The incidence of protozoan parasitic infection was higher in the rainy Season (August to December) than in the dry Season (April to June) (32.7% and 2.7%, respectively) ( Table 4).

**Table 2.** Frequency of protozoan pathogens in children with diarrhea in Khartoum among the age.

	Age in years			P-value
	0-2	2-4	4-5	
***G. lamblia***	17.2%	1.6%	0%	0.715
***Cryptosporidium parvum***	14.6%	1.1%	0%	0.828
***E. histolytica***	0.9%	0%	0%	0.843
**Total**	32.7%	2.7%	0%	

**Table 3.** Frequency of protozoan pathogens in children with diarrhea in Khartoum among the gender.

	Gender		P-value
	M	F	
***G. lamblia***	14.2%	4.6%	0.010
***Cryptosporidium parvum***	9.4%	6.4%	0.483
***E. histolytica***	0.2%	0.7%	0.112
**Total**	23.8%	11.7%	

**Table 4.** Frequency of protozoan pathogens in children with diarrhea in Khartoum among the Seasons.

	Season		P-value
	Autumn	Summer	
***G. lamblia***	16.9%	1.8%	0.391
***Cryptosporidium parvum***	14.9%	0.9%	0.064
***E. histolytica***	0.9%	0%	0.446
**Total**	32.7%	2.7%	

### Parasitic mono- and co-infections

Infection with monoparasite was found in 139 cases (31.8%). In contrast, parasite co-infection was detected in 16 patients (3.7%), which involved *E. histolytica* and *G. lamblia* in two cases, and *G. lamblia* and *C. parvum* in 14 cases ( Table 5).

**Table 5.** Frequency of samples with co-infections.

Pathogen	No. of co-infections (%)
*G. lamblia* and *C. parvum*	14 (3.2%)
*E. histolytica* and *G. lamblia*	2 (0.5%)
Total	16 (3.7%)

## Discussion

Gastrointestinal protozoan parasites still pose common health problems, mainly in children aged less than 5 years worldwide. The rapid and accurate identification of protozoan parasites is a big challenge in many developing countries. The real-time multiplex PCR technique that provides concurrent detection of all protozoal parasites used herein was an exceedingly powerful laboratory system, enabling rapid, sensitive, precise, and inexpensive parasite detection.

This study was conducted during two seasons (autumn and summer), from April to June and August to December 2014, in Khartoum State, Sudan. The present study aimed to determine the prevalence of certain protozoan parasites linked with acute gastroenteritis in stool samples from children under five years old using a multiplex real-time PCR assay developed in a previous study. ^11^

Among the 437 fecal specimens examined, 276 were collected from male children and 161 from females, amounting to a male-to-female ratio of 1:1.7. Most of the samples were from less than ≤2 years old (403, 92.2%), followed by >2–≤4 years (32, 7.3%) and >4–˂5 years (2, 0.5%).

As they showed in Table 2, the gender distribution among the *G. lamblia*-positive samples was 14.2% in males and 4.6 % in females (P<0.01), indicating a statistically significant difference among the gender group that supports the statement of Khwam H. ^12^

The most significant number of samples were from the age group ≤2 years (403, 92.2%), followed by >2–≤4 years (32, 7.3%) and >4–˂5 years (2, 0.5%). The result of our study indicates the highest Prevalence of protozoal diarrhea (32.7%) was detected in the age group of ≤2 years flowed by >2–≤4 years (2.7%), and no protozoan pathogen was found in the age group of >4–˂5 years; however, these results could be explained by the fact that most of our samples were collected from the age group ≤2 years in which the decline of the maternal immunity with an age risk factor of diarrhea infection. ^13^^,^^14^ The highest positivity was detected in the samples of boys less than two years old. The reason that the numbers of children with diarrhea (boys of ≤2 years) admitted to hospitals is not apparent, and more research is needed to determine whether this is the pattern of childhood diarrhea in Sudan. The contaminated hands and bad hygiene may contribute to the transmission of food borne infection in these children, which was in agreement with the investigation in Nepal, where the highest Prevalence of parasitic diarrhea was found in the age group of fewer than two years. ^15^However,our result differed from another study by Saeed *et al*. in Khartoum ^16^ in which the major group of infections was in >4–˂5 years old, and this may again be due to statistical bias since most of samples were collected from the age group of ≤2 years; This should be investigated in future studies by using larger sample size in different Season.

The result revealed a higher prevalence (35.5%) of protozoan diarrhea disease compared with other studies conducted in Khartoum state (16%). ^16^ and in other developing countries, including Nepal and Ethiopia (0.7%, 15.6%, respectively). ^15^^,^^17^ In comparison, the incidence was lower than that in Tanzania and South Africa (55.6%, 68%, respectively) ^18^^,^^19^and close to that reported in the Gaza strip (39%). ^20^ Our study is the first to demonstrate a high Prevalence of *Cryptosporidium parvum* (15.8%) in Sudan. The diagnosis of *Cryptosporidium* used to depend on the Ziehl−Neelsen stain, and it was neglected mainly by our laboratories until we used a sensitive molecular assay that increased the detection rate of these agents.

The most prevalent protozoan detected in the present investigation was *G. lamblia*, with a prevalence of 18.8%, which is higher than in the study conducted in Khartoum State (15.8%). ^16^ Its Prevalence was followed by *C. parvum* (15.8%) and *E. histolytica* (0.9%). This result was consistent with previous findings in developing countries, including India, Gaza and Nigeria. ^13^^,^^20^^,^^21^ Infection with mono protozoan parasite was found in 139 cases (31.8%) cases; co-infection was found in 16 cases (3.7%). The study of co-infection on clinical severity was not studied in these patients. However, it has been reported that no significant variation was reported in the clinical symptoms of patients with co-infections compared with those with single infections. ^22^ Our study showed that the incidence of a protozoan parasite is higher in autumn (wet) than that in summer (dry), which was in accordance with the study conducted in Khartoum state. ^16^ It should be noted that no protozoan pathogen was detected in stool samples,which were likely due to infections with other pathogens like viruses and bacteria and also may be due to noninfectious reasons like hypersensitivity to certain food ingredients and weaning diarrhea that result of the inability of an underdeveloped child intestine to metabolize the food. Poor hygiene and sanitation and lack of proper toilets may facilitate these infections.

The present study furnished some crucial insights into the protozoan cause of child diarrhea in Khartoum State. Findings this study will help us understand the importance of parasitic diarrhea in children younger than five years, and these findings are valuable in developing measures to enhance the health condition of the young children. Furthermore, this study calls for the establishment of sensitive and specific molecular techniques, such as multiplex PCR, for the detection of the protozoan pathogen in a clinical setting, which is essential.

## Authors contributions

Mosab, Isam, and Xuejun designed the experiment; Mosab and Hong-do the lab experiment; Azza analyzed the data; Mosab, Khalid, and Abdel collected the samples. Mosab Hong and Abdel wrote the article.

## Data Availability

Excel, https://doi.org/10.6084/m9.figshare.21201676.v3. ^23^ This project contains the following underlying data:
•
Data information.xlsx The material is available under the terms of the Creative Commons Attribution 4.0 International license (CC-BY 4.0).
