# Molecular survey of certain protozoan agents that cause diarrhea in children in Sudan

**DOI:** 10.12688/f1000research.123652.3

**Published:** 2024-06-14

**Authors:** Mosab Adam, Hongwei Shen, Khalid-A Enan, Hao Wang, Azza B. Musa Musa, Abdel R. El Hussein, Isam M. Khidir, Xuejun Ma

**Affiliations:** 1 Department of Virology, Ministry of Higher Education and Scientific Research, Khartoum, Sudan,, khartoum, Sudan; 2 National Institute for Viral Disease Control and Prevention, Chinese Center for Disease Control and Prevention, China., beijing, China; 3 Futian District Center for Disease Control and Prevention, Shenzhen, China., Shenzhen, China; 4 Department of Microbiology and Parasitology,, Faculty of Medicine, University of Khartoum, Khartoum, Sudan, khartoum, Sudan

**Keywords:** Diarrhea, Detection, Parasitic, Protozoan, Pathogens, Childhood

## Abstract

**Introduction:**

Diarrhea is a significant health problem in the Third World. Identification of the pathogen that causes diarrhea is vital for measures to prevent and control this disease. There are also very few reports of diarrhea in Sudan. Our study aimed to determine the Prevalence of specific protozoan pathogens ( *Entamoeba histolytica*, *Cryptosporidium* parvum., and *Giardia spp*) in children in Khartoum, Sudan.

**Methods:**

We conducted a cross-sectional survey among children under five years of age who were hospitalized with acute diarrhea between April and December 2014. Diarrheal stool samples were collected and *E. histolytica, C. parvum, and Giardia spp were examined* using multiplex real-time PCR.

**Results:**

Four hundred and thirty-seven children with acute diarrheawere included in this study; the higher Prevalence of diarrhea was in the age less than ≤ 2 years old (403,92.2%). The male-to-female ratio in this study was 1:1.7. infection with intestinal parasite was found in 155 (35.5%) cases, and co-infection was detected in 16 (10.3%) cases. *Giardia spp*(18.8%) and *C. parvum* (15.8 %) were the most frequently identified parasites, followed by *E. histolytica* (0.9). The parasite infection rate was highest and lowest in the under 2-year-old group (92.3%) and the 2–4-year-old group (7.3%). The infection rate was higher in boys (67.1%) than in girls (32.9%). The incidence of protozoan infection was higher in the rainy season (August to December) (92.2%), corresponding with that in the dry Season (April to June). (7.8%).

**Discussion:**

Our present study demonstrated the high prevalence of *Giardia spp* and *C. parvum* in children with diarrhea in the Khartoum region and the usefulness of the multiplex real-time method in disclosing pathogenic protozoal agents. Our result highlighted the necessity of developing intervention measurement and control strategies to deal with childhood parasitic diarrhea in this region.

## Introduction

Diarrhea is defined as passing soft, loose, or watery feces three times or more in 24 hours; it is usually a result of the consumption of pathogen-contaminated water or food. ^1^ Diarrhea remains the leading cause of death and illness in children in third-world countries. ^2^^–^^4^ Around 1.7 billion cases of childhood diarrhea are reported each year, and diarrhea is estimated to have killed more than 500,000 children under the age of 5 worldwide in 2019. ^1^^,^^5^ Where diarrhea is considered the third most common cause for young children to visit health centers, some of the underlying conditions found in the community of most developing countries, including malnutrition and poor hygiene, may increase the risk of experiencing diarrheal disease. ^6^ In developed countries, the availability of modern technologies and suitable water supply has led to a decline in global death due to diarrhea; however, despite the substantial effort to supply modern technology and management practices, diarrhea in Africa is still unacceptably ranked as the second cause of death among young children. ^7^^–^^10^ Despite the high morbidity of childhood diarrhea in Sudan, the knowledge of the parasitic causative agents is scant. Parasitic protozoans that infect the intestinal tract in developing countries include *Cryptosporidium spp.* *Giardia spp.*, and *Entamoeba histolytica*, respectively, the agents that cause cryptosporidiosis, giardiasis, and amoebiasis, which are considered prime for diarrheal diseases in children under 5 years old. ^11^ The limited specificity and sensitivity of the microscopic method commonly used in most Sudan laboratories decreased the parasitic infection detection rate. As a result, there is little information about the precise incidence of diarrhea and causative protozoan agents.

This study aimed to explore the incidence of some protozoan organisms ( *Cryptosporidium parvum*, *Giardia spp*., and *Entamoeba histolytica*) that produce acute diarrheal illness among young children using molecular techniques.

## Methods

### Ethical considerations

The study was approved by the ethical committee of the Sudan Academy of Sciences (Approval number 2367), and written permission was obtained from the registered child’s parents or guardian.

### Design, area, and period of study

This cross-sectional study was co-conducted at the Central Laboratory, Ministry of Higher Education and Research, Sudan, and the National Institute for Viral Disease Control and Prevention, China Center for Disease Control and Prevention, China (CDC), Beijing, China, during two different Seasons (the hot, dry Season from April to June (Summer), and the rainy Season from August to December (Autumn) in the year 2014 at Khartoum teaching hospitals.

### Participants (inclusion and exclusion criteria)

A total of 437 fecal samples (one per patient) were collected from children admitted to hospitals who had been clinically diagnosed with acute diarrhea from 1 to 4 days before the sample collection, were less than five years old, and mostly lived in a rural area.

### Sample collection and storage

The stool sample was collected in a dry, clean plastic container. The specimens were kept at −20°C till tested in early 2015; frozen samples were sent via dry ice to the Center for Disease Control and Prevention in Beijing, China.

### Data collection

Patient data including age, sex, and Season, were collected through a structured questionnaire.

### Nucleic acid extraction

Per the manufacturer’s instructions, parasite DNA was extracted from 200 μL of 10% fecal suspension prepared in phosphate buffer saline using QIAamp ^®^ Fast DNA Stool Mini Kit (Qiagen, Hilden, Germany). The extracts were eluted in 60 μL of DNase-free water, immediately aliquoted in 20 μL, and kept at −80°C.

### PCR amplification and parasite detection

**Primers and probes of multiplex real-time PCR**

Three primer pairs and three probes were used for the simultaneous detection of E. histolytica, C. parvum, and Giardia *spp*. ^11^ Table 1 shows the oligonucleotide sequence of the primers, probes, and target genes.

**Table 1.** The nucleotide primers and probes for multiplex real-time PCR were used in this study.

Organism	Target gene ^a^	Primer sequence (5′-3′) ^b^	Probe (5′-3′)
*E. histolytica*	SSU rRNA	F: ATTGTCGTGGCATCCTAACTCA R: GCGGACGGCTCATTATAACA	VIC-TCATTGAATGAATTGGCCATTT ^11^
*Giardia spp.*	SSU rRNA	F: GACGGCTCAGGACAACGGTT R: TTGCCAGCGGTGTCCG	FAM-CCCGCGGCGGTCCCTGCTAG ^11^
*C. parvum*	DNA-like	F: CGCTTCTCTAGCCTTTCATGA R: CTTCACGTGTGTTTGCCAAT	Texas Red-CCAATCACAGAATCA ^11^ TCAGAATCGACTGGTATC ^11^

^a^ SSU rRNA, small subunit ribosomal RNA.

^b^ F, forward; R, reverse.

**Multiplex real-time PCR**

Real-time PCR was performed with a Multiplex PCR kit (Qiagen, Hilden, Germany) in a 20 μL volume containing 6.25 pmol of each *E. histolytica-*specific primers, 6.25 pmol of each *Giardia spp.*-specific primers, 25 pmol of each *C. parvum*-specific primers, 1.75 pmol of *E. histolytica*-specific VIC-TaqMan probe, 2.5 pmol of *G. lamblia*-specific FAM-TaqMan probe, 8.75 pmol of *C. parvum*-specific Texas Red-TaqMan probe. Amplification consisted of 15 min at 95°C, 40 cycles of 15 s at 95°C, 30 s at 60°C, and 30 s at 72°C. The iCycler real-time detection system (Bio-Rad) performed amplification, detection, and data analysis.

### Statistical analysis

The Chi-square test assessed differences in proportions. P values <0.05 were considered statistically significant.

## Result

### Demographic data on the population

A total of 437 children admitted to hospitals had been clinically diagnosed with acute diarrhea, Comprising 276 boys and 161 girls. The participants were aged ≤2 years (403, 92.2%), >2–≤4 years (32, 7.3%), and >4–˂5 years (2, 0.5%).

### Prevalence of parasitic infections

**Diagram 1.**
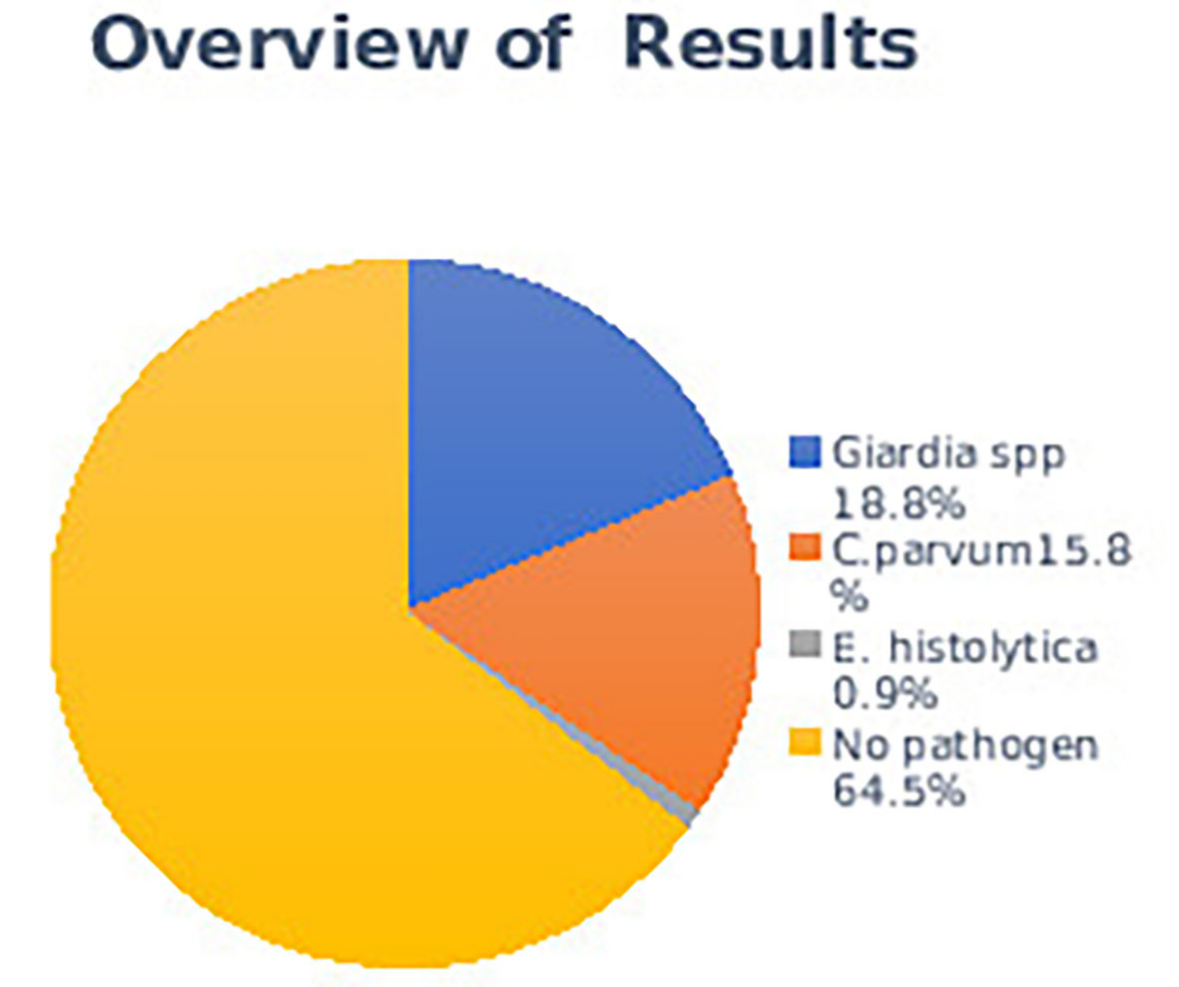
Overview of protozoal parasite infections.

Protozoal were diagnosed in 155/437 (35.5%) cases, among which the highest prevalence was *Giardia spp.* (82/437, 18.8%), followed by *C. parvum* (69/437, 15.8%), and *E. histolytica* (4/437, 0.9%). None of them were detected in 282 (64.5%) diarrheal cases ( Diagram 1).

### Distribution of parasitic infections by age, sex, and seasonality

The highest rate of parasitic infection was seen in the ≤2 years group (92.3%) and much lower in the >2–≤4 years old group (7.7%) ( Table 2). In contrast, the protozoal parasite was not detected in the age group of >4–˂5 years. Among children with parasitic infections, 104(67.1%) were male, while 51(32.9%) were female ( Table 3). Diarrhea remains the leading cause of illness in children ≤2 years (403, 92.2%). The incidence of protozoan parasitic infection was higher in the rainy Season (August to December) than in the dry Season (April to June) (143(92.2%) and 12(7.8%), respectively) ( Table 4).

**Table 2.** Frequency of protozoan pathogens in children with diarrhea in Khartoum among the age.

	Positive	Age in years			P-value
	N	0-2	2-4	4-5	
***Giardia spp.***	82 (18.8%) *	75 (48.4%)	7 (4.5%)	0 (0%)	0.715
***C. parvum***	69 (15.8%) *	64 (41.3%)	5 (3.2%)	0 (0%)	0.828
***E. histolytica***	4 (0.9%) *	4 (2.6%).	0 (0%)	0 (0%)	0.843
**Total**	155 (35.5%) *	143 (92.3%)	12 (7.7%)	0%	

* Percent inside curly bracket found on the second row and column are % out of 437 patients.

**Table 3.** Frequency of protozoan pathogens in children with diarrhea in Khartoum among the sex.

	Positive	Sex		P-value
	N	M	F	
***Giardia spp.***	82 (18.8%) *	62 (40%)	20 (12.9%)	0.010
***C. parvum***	69 (15.8%) *	41 (26.5%)	28 (18.1%)	0.483
***E. histolytica***	4 (0.9%) *	1 (0.6%)	3 (1.9%)	0.112
**Total**	155 (35.5%) *	104 (67.1%)	51 (32.9%)	

* Percent inside curly bracket found on the second row and column are % out of 437 patients.

**Table 4.** Frequency of protozoan pathogens in children with diarrhea in Khartoum among the Seasons.

	Positive	Season		P-value
	N	Autumn	Summer	
***Giardia spp.***	82 (18.8%) *	74 (47.7%)	8 (5.1%)	0.391
***C. parvum***	69 (15.8%) *	65 (41.9%)	4 (2.6%)	0.064
***E. histolytica***	4 (0.9%) *	4 (2.6)	0 (0%)	0.446
**Total**	155 (35.5%) *	143 (92.2%)	12 (7.8%)	

* Percent inside curly bracket found on the second row and column are % out of 437 patients.

### Parasitic mono- and co-infections

Infection with monoparasite was found in 139 cases (89.7%). In contrast, parasite co-infection was detected in 16 patients (10.3%), which involved *E. histolytica* and *Giardia spp.* in two cases, and *Giardia spp.* and *C. parvum* in 14 cases ( Table 5).

**Table 5.** Frequency of samples with co-infections.

Pathogen	No. of co-infections (%)
*Giardia spp.* and *C. parvum*	14 (9%) *
*E. histolytica* and *Giardia spp.*	2 (1.3%) *
Total	16 (10.3%) *

* Percent inside curly bracket found on the second row and column are % out of 155 patients.

### Statistical analysis

The occurrence of *Giardia spp.* in male children was statistically significant than in females (P < 0.01); besides that, the comparisons between these variables were not relevant ( Tables 2, 3, 4).

## Discussion

Gastrointestinal protozoan parasites still pose common health problems, mainly in children under five years worldwide. Rapid and accurate identification of protozoan parasites is a big challenge in many developing countries. The real-time multiplex PCR technique used herein, which provides concurrent detection of all protozoal parasites, is an exceedingly powerful laboratory system, enabling rapid, sensitive, precise, and inexpensive parasite detection.

The present study aimed to determine the prevalence of certain protozoan parasites linked with acute gastroenteritis in stool samples from children under five years old using a multiplex real-time PCR assay developed in a previous study. ^11^

As shown in Table 2, the sex distribution among the *Giardia spp.*-positive samples was 67.1% in males and 32.9% in females (P<0.01), indicating a statistically significant difference among the sex group that supports the statement of Khwam H. ^12^

The most significant number of samples were from the age group ≤2 years followed by >2–≤4 years and >4–˂5 years. The result of our study indicates the highest Prevalence of protozoal diarrhea was detected in the age group of ≤2 years followed by >2–≤4 years and no protozoan pathogen was found in the age group of >4–˂5 years; however, these results could be explained by the fact that most of our samples were collected from the age group ≤2 years in which the decline of the maternal immunity with an age risk factor of diarrhea infection. ^13^^,^^14^ The highest positivity was detected in the samples of boys less than two years old. The reason that the number of children with diarrhea (boys of ≤2 years) admitted to hospitals is not apparent, and more research is needed to determine whether this is the pattern of childhood diarrhea in Sudan. The contaminated hands and bad hygiene may contribute to the transmission of food-borne infection in these children, which was in agreement with the investigation in Nepal, where the highest Prevalence of parasitic diarrhea was found in the age group of fewer than two years. ^15^ However,our result differed from another study by Saeed *et al*. in Khartoum ^16^ in which the major group of infections was >4–˂5 years old, and this may again be due to statistical bias since most of the samples were collected from the age group of ≤2 years; This should be investigated in future studies by using larger sample size in different Season.

The result revealed a higher prevalence (35.5%) of protozoan capable of causing children diarrhea compared with other studies conducted in Khartoum state (16%). ^16^ and other developing countries, including Nepal and Ethiopia (0.7% and 15.6%, respectively). ^15^^,^^17^ In comparison, the incidence was lower than that in Tanzania and South Africa (55.6% and 68%, respectively) ^18^^,^^19^ and close to that reported in the Gaza Strip (39%). ^20^ Our study is the first to demonstrate a high Prevalence of *Cryptosporidium parvum* (15.8%) in Sudan. The diagnosis of *Cryptosporidium* used to depend on the Ziehl−Neelsen stain, and it was neglected mainly by our laboratories until we used a sensitive molecular assay that increased the detection rate of these agents.

The most prevalent protozoan detected in the present investigation was *Giardia spp.*, with a prevalence of 18.8%, higher than in the study conducted in Khartoum State (15.8%). ^16^ Its Prevalence was followed by *C. parvum* (15.8%) and *E. histolytica* (0.9%). This result was consistent with previous findings in developing countries, including India, Gaza, and Nigeria. ^13^^,^^20^^,^^21^ Infection with mono protozoan parasite was found in 139 cases (89.7%) cases; co-infection was found in 16 cases (10.3%). The study of co-infection on clinical severity was not studied in these patients. However, it has been reported that no significant variation was reported in the clinical symptoms of patients with co-infections compared with those with single infections. ^22^ Our study showed that the incidence of a protozoan parasite is higher in autumn (wet) than with summer (dry) which was under the study conducted in Khartoum state. ^16^ It should be noted that no protozoan pathogen was detected in stool samples, which were likely due to infections with other pathogens like viruses and bacteria and also may be due to noninfectious reasons like hypersensitivity to certain food ingredients and weaning diarrhea that result from the inability of an underdeveloped child intestine to metabolize the food. Poor hygiene and sanitation and lack of proper toilets may facilitate these infections. ^16^

## Conclusion

The current investigation provided some vital information about the protozoan source of diarrhea in children in the state of Khartoum.

These findings are valuable in developing measures to improve the health condition of the young. Furthermore, this study calls for establishment of sensitive and specific molecular methods, such as multiplex PCR, for detecting the protozoan pathogen in a clinical setting, which is essential.

## Authors contributions

Mosab, Isam, and Xuejun designed the experiment; Mosab and Hong performed the lab experiment; Azza analyzed the data; Mosab, Khalid, and Abdel collected the samples; and Mosab Hong and Abdel wrote the article.

## Data Availability

Excel, https://doi.org/10.6084/m9.figshare.21201676.v3. ^23^ This project contains the following underlying data:
•
Data information.xlsx The material is available under the terms of the Creative Commons Attribution 4.0 International license (CC-BY 4.0).
