# Supplementary material for: Molecular survey of certain protozoan agents that cause diarrhea in children in Sudan
Source: F1000Res. 2024 Oct 23;11:1401. Originally published 2022 Nov 29. [Version 4] doi: 10.12688/f1000research.123652.4 (PMC11325136; doi:10.12688/f1000research.123652.4)
Supplement: Peer review history

# Reviewer report: Reviewer response for version 4

**DOI:** 10.5256/f1000research.173182.r334246

**Published:** 2024-11-04

**Authors:** Lawrence R. Schiller

**Affiliations:** 1 Baylor Scott & White Health, Dallas, Texas, USA

This observational study has limited generalizability or interest beyond the geographic region involved.

Is the work clearly and accurately presented and does it cite the current literature?

Partly

If applicable, is the statistical analysis and its interpretation appropriate?

Partly

Are all the source data underlying the results available to ensure full reproducibility?

Partly

Is the study design appropriate and is the work technically sound?

Partly

Are the conclusions drawn adequately supported by the results?

Partly

Are sufficient details of methods and analysis provided to allow replication by others?

Yes

Reviewer Expertise:

Diarrheal disease

I confirm that I have read this submission and believe that I have an appropriate level of expertise to confirm that it is of an acceptable scientific standard.

# Reviewer report: Reviewer response for version 3

**DOI:** 10.5256/f1000research.167575.r291316

**Published:** 2024-09-09

**Authors:** Francisco Ponce-Gordo

**Affiliations:** 1 Complutense University of Madrid, Madrid, Spain

The authors have improved the article, however there are some points that should be corrected. The new consideration as "not approved" is based on the errors in the calculation of the percentage values for statistical analyses.

 1. Presentation. There are typographical and or grammar errors in the text. To mention only a few, in abstract: in introduction, please write "parvum" in italics; in methods, " *spp. were examined*" should not be in italics; in results, in the first line, place a space in "diarrheawere", write 'Prevalence' in lower case, correct "age less than ≤ 2 years" to "age less than 2 years" or "age ≤ 2 years", correct the number (403,92.2%) or indicate what is each number, ...

 In other cases, expressions are incorrect or have no sense; i.e., in discusion, paragraph #6: "infection with mono protozoan parasite was found ...", In this case, "single protozoan infections" would best be used.

 There are many small corrections to do.

 2. Content.

 In abstract-results, "the incidence of protozoan infection ...". Change to "the number of positive cases ...". Incidence is the number of new cases in the population, and prevalence the number of infected persons in the population. In both cases, the population should be known (the total population, not the total number of symptomatic patients included in the study).

 In methods-participants (inclusion and excllusion criteria): say only the conditions for inclusion/exclusion; the number of patients accepted will be provided in results. Also, "mostly lived" is not a criterion for inclusion/exclusion (the criterion for acceptance/rejection should be 'lived' or 'not lived' in a rural area)

 In methods - table 1: what is target gene: DNA-like? "DNA-like" is not a gene.

 Methods-statistical analysis: please indicate which variables were included in the analysis. As proportions were in some cases incorrectly calculated (see below), please revise the analysis and re-do.

 In results-distribution of parastic infections by age, sex and seasonality: in order to keep consistency with the data provided in the previous subsection, please use the same format for each group: positive number/total number of samples (%)

 In this same section, the fourth sentence ("Diarrhea remains ..." has no sense.The samples were collected from symptomatic patients, so all of them were diarrheic or, at least, the patients had diarrhea in the previous days. It cannot be said that "diarrhea remains the leading cause of illness in children", the data presented do not support this. This might be a comment for the discusion section, if adequate references are provided.

 In diagram 1: "no pathogen" is not correct, please use "no protozoa". In those patients, diarrhea could have been caused by other pathogens as viruses or bacteria.

 In tables 2-4: the percentages seem to be incorrectly calculated. For each category (i.e., for 0-2 years, 2-4 years, 4-5 years; for male and for females; and for autumn and summer), the percentage should be calculated as the number of positive cases/total number of cases in THE CATEGORY, not in the total. That is, for example in *Giardia *and the values in the 0-2 years category: 75 positive samples (in that category) respect to 403 samples (the number of 0-2 years samples analyzed), is 18.6% (not 48.4%, which has been calculated as the percentage of positive samples at that category, respect to the total number of positive samples for all categories and all parasites). All the percentages are incorrect, and the statistical results are also incorrect, this affecting the results and the discusion sections. Please provide the number of total cases for each category, and re-calculate the percentages accordingly.

 In statistical analysis: it should be re-done. In the revised version, please indicate the results for all protozoa, not only one of them (as in the present case, only for *Giardia*). The sentence that it is now in the text is also incorrect, it should be something like "the occurrence of *Giardia *respect to sex is statistically signifcant, being greater in males than in females" or as "the occurrence of *Giardia *in male children was higher and statistically significant than in females".

 In discusion, ending of the first paragraph, "sensitive": what about false negatives? Please provide references to support the statements.

 Also in discusion: avoid paragraphs consisting on single sentences. Place the second paragraph at the end of the first one.

 Discusion should be redone according to the new results from the statistical analysis. The geneal structure of the section followed now can be kept. Avoid repeating results (i.e., in the third paragraph, "As shown in table 2, ..." ); also, this paragraph would be best moved and included with the text dealing with *Giardia*.

 In discusion, paragraph #6, "It should be noted that no protozoan pathogen was detected in stool samples". If this sentence applies to the reference just cited in the previous sentence (a study conducted in Khartoum state), please clearly state it. In the present study, some positive protozoan infections were detected in summer samples.

 In conclusion: "the current investigation provided some vital information ...". The information provided in the manuscript is not of vital importance, please moderate the tone. The study focuses on the parasites that cause diarrhea, and these parasites are well-known pathogens. If the data were on the source of the infections (water or food), it would be very important as it will allow adopting preventive measures to avoid further infections.

Is the work clearly and accurately presented and does it cite the current literature?

Partly

If applicable, is the statistical analysis and its interpretation appropriate?

Partly

Are all the source data underlying the results available to ensure full reproducibility?

Yes

Is the study design appropriate and is the work technically sound?

Yes

Are the conclusions drawn adequately supported by the results?

Partly

Are sufficient details of methods and analysis provided to allow replication by others?

Yes

Reviewer Expertise:

Parasitology - Protozoology - molecular taxonomy

I confirm that I have read this submission and believe that I have an appropriate level of expertise to state that I do not consider it to be of an acceptable scientific standard, for reasons outlined above.

# Response

**Published:** 2024-10-08

**Authors:** Mosab Adam

**Affiliations:** Central laboratory -Sudan, Sudan

We appreciate the insightful feedback provided, which has significantly enhanced the quality of our manuscript. In this revised version, we have incorporated your comments and suggestions. The key modifications include:

 - Corrections made throughout the manuscript, including adjustments to the table and figure, as indicated by track changes.

 - Updates to the percentages in all tables to reflect the appropriate categories.

 - Revisions to the figure in accordance with the reviewers' recommendations.

 - A thorough review for typographical and grammatical errors.

 -We revise the reference position in the manuscript and update the related references accordingly.

 In response to your comments, we have addressed the concerns raised and reanalyzed the statistical results accordingly.

# Reviewer report: Reviewer response for version 3

**DOI:** 10.5256/f1000research.167575.r291313

**Published:** 2024-08-14

**Authors:** Monira Sarmin

**Affiliations:** 1 International Centre for Diarrhoeal Disease Research, Dhaka, Bangladesh

The authors did the necessary revision. This version is more acceptable. Statistical section under methodology needs more elaboration.

Is the work clearly and accurately presented and does it cite the current literature?

Partly

If applicable, is the statistical analysis and its interpretation appropriate?

No

Are all the source data underlying the results available to ensure full reproducibility?

Partly

Is the study design appropriate and is the work technically sound?

Yes

Are the conclusions drawn adequately supported by the results?

Partly

Are sufficient details of methods and analysis provided to allow replication by others?

Partly

Reviewer Expertise:

NA

I confirm that I have read this submission and believe that I have an appropriate level of expertise to confirm that it is of an acceptable scientific standard.

# Reviewer report: Reviewer response for version 3

**DOI:** 10.5256/f1000research.167575.r291317

**Published:** 2024-07-02

**Authors:** Lawrence R. Schiller

**Affiliations:** 1 Baylor Scott & White Health, Dallas, Texas, USA

This paper reviews data collected 10 years ago. The patient sample was a convenience sample--patients admitted to hospital with acute diarrhea. This sample may not reflect prevalence of protozoal diarrhea in the larger population in Sudan. It only reflects the findings in patients so sick as to require hospitalization because of volume depletion or other complications of diarrhea. The discussion needs to be modified to mention this; the prevalence of protozoal diseases as a proportion of all diarrheal diseases may be impacted by the occurrence of outbreaks of other diarrheal diseases. Were there any concurrent outbreaks (e.g., cholera, E.coli, etc.) in 2014?

 If the authors want to assess the prevalence of protozoal diarrhea in children in Sudan as the title indicates, the background carriage in asymptomatic children and the prevalence in children with diarrhea who are not hospitalized should be checked.

Is the work clearly and accurately presented and does it cite the current literature?

Partly

If applicable, is the statistical analysis and its interpretation appropriate?

Partly

Are all the source data underlying the results available to ensure full reproducibility?

Partly

Is the study design appropriate and is the work technically sound?

Partly

Are the conclusions drawn adequately supported by the results?

Partly

Are sufficient details of methods and analysis provided to allow replication by others?

Yes

Reviewer Expertise:

Diarrheal disease

I confirm that I have read this submission and believe that I have an appropriate level of expertise to state that I do not consider it to be of an acceptable scientific standard, for reasons outlined above.

# Reviewer report: Reviewer response for version 2

**DOI:** 10.5256/f1000research.161677.r271108

**Published:** 2024-06-06

**Authors:** Sonia Boughattas

**Affiliations:** 1 Qatar University, Doha, Doha, Qatar

This work is of interest in the field with clear workflow and understandable impact.

 However, several points need to be taken in consideration

 -Language editing is required especially for the abstract and introduction parts

 -G. lamblia name is no longer frequently used, please replace by G. intestinalis

 -Within the Results section, different points need to be addressed:

 i) I don’t believe the mention of “undiagnosed cases” is right. Same with the statement “No protozoan pathogen was detected in 282 (64.5%) diarrheal cases (Diagram 1).”

 These cases can be due to other protozoan pathogens not screened in the current study as Blastocystis sp. Dientamoeba fragilis or even other Cryptosporidium and Entamoeba species.

 I would suggest removing Diagram 1 and report only to the targeted protozoa prevalence.

 ii) The statically significant p-value should be mentioned in Bold in the tables

 -The discussion part lacks some sections: the difference in prevalence between genders within Giardia is statistically significant so this factor needs further comparisons and discussion.

 Same for the season factor, an explanation should be provided according to t the literature on why high prevalences of protozoa are observed within the Wet season compared to the Dry season.

 Once the different points are amended, the manuscript quality will be improved and thus be indexed.

Is the work clearly and accurately presented and does it cite the current literature?

Partly

If applicable, is the statistical analysis and its interpretation appropriate?

Partly

Are all the source data underlying the results available to ensure full reproducibility?

Yes

Is the study design appropriate and is the work technically sound?

Yes

Are the conclusions drawn adequately supported by the results?

Partly

Are sufficient details of methods and analysis provided to allow replication by others?

Yes

Reviewer Expertise:

Molecular epidemiologu, Protozoa, Zoonosis

I confirm that I have read this submission and believe that I have an appropriate level of expertise to confirm that it is of an acceptable scientific standard, however I have significant reservations, as outlined above.

# Response

**Published:** 2024-06-07

**Authors:** Mosab Adam

**Affiliations:** Central laboratory -Sudan, Sudan

Thanks for the valuable comments. That will surely increase the quality of our manuscript, so for now, I have submitted version 3, which is being processed with a typesetter and will be available online soon. Fortunately, in this version, we addressed most of the points you mentioned( G.lamblia, “undiagnosed cases statement.) Which was also recommended by a past reviewer. Later, we will change the others. I wish to recheck it when it becomes available online 

 Best regards 

 Mosab adam

# Reviewer report: Reviewer response for version 2

**DOI:** 10.5256/f1000research.161677.r271112

**Published:** 2024-05-10

**Authors:** Lawrence R. Schiller

**Affiliations:** 1 Baylor Scott & White Health, Dallas, Texas, USA

This paper reviews data collected 10 years ago. The patient sample was a convenience sample--patients admitted to hospital with acute diarrhea. This sample may not reflect prevalence of protozoal diarrhea in the larger population in Sudan. It only reflects the findings in patients so sick as to require hospitalization because of volume depletion or other complications of diarrhea. The discussion needs to be modified to mention this; the prevalence of protozoal diseases as a proportion of all diarrheal diseases may be impacted by the occurrence of outbreaks of other diarrheal diseases. Were there any concurrent outbreaks (e.g., cholera, E.coli, etc.) in 2014?

 If the authors want to assess the prevalence of protozoal diarrhea in children in Sudan as the title indicates, the background carriage in asymptomatic children and the prevalence in children with diarrhea who are not hospitalized should be checked.

Is the work clearly and accurately presented and does it cite the current literature?

Partly

If applicable, is the statistical analysis and its interpretation appropriate?

Partly

Are all the source data underlying the results available to ensure full reproducibility?

Partly

Is the study design appropriate and is the work technically sound?

Partly

Are the conclusions drawn adequately supported by the results?

Partly

Are sufficient details of methods and analysis provided to allow replication by others?

Yes

Reviewer Expertise:

Diarrheal disease

I confirm that I have read this submission and believe that I have an appropriate level of expertise to state that I do not consider it to be of an acceptable scientific standard, for reasons outlined above.

# Reviewer report: Reviewer response for version 2

**DOI:** 10.5256/f1000research.161677.r251189

**Published:** 2024-05-07

**Authors:** Monira Sarmin

**Affiliations:** 1 International Centre for Diarrhoeal Disease Research, Dhaka, Bangladesh

The manuscript needs a few corrections.

 The following things should be addressed before finalization.

 1.Abstract: Results section needs revision:       

 1st line- 437 acute children were included, what does acute children mean?

 2.The parasite infection rate was highest and lowest in the under 2-year-old group (32.7%), and in the 2–4-year-old group (2.7%), the infection rate was higher was higher in boys (23.7%) than in girls (11.7%). The incidence of protozoan infection was higher (37.7%) in the rainy season (August to December) (32.7%), corresponding with that in the dry Season (April to June). (2.7%).

 This section needs revision, difficult to apprehend it.

 3.Data collection: Patient data including age, biological gender, and Season, were collected through a structured questionnaire.

 Actually, we record sex of the child not the gender.

 4.Results: Table 3- probably the authors assessed sex not the gender.

Is the work clearly and accurately presented and does it cite the current literature?

Partly

If applicable, is the statistical analysis and its interpretation appropriate?

No

Are all the source data underlying the results available to ensure full reproducibility?

Partly

Is the study design appropriate and is the work technically sound?

Yes

Are the conclusions drawn adequately supported by the results?

Partly

Are sufficient details of methods and analysis provided to allow replication by others?

Partly

Reviewer Expertise:

NA

I confirm that I have read this submission and believe that I have an appropriate level of expertise to confirm that it is of an acceptable scientific standard, however I have significant reservations, as outlined above.

# Response

**Published:** 2024-06-09

**Authors:** Mosab Adam

**Affiliations:** Central laboratory -Sudan, Sudan

I appreciate your efforts and comments that sure will increase the quality of my manuscript 

 we corrected the points you mentioned in your review

 - we change gender to sex in the text and table 

 -We corrected the sentence you said in the abstract. (This section needs revision; it is difficult to comprehend.)

# Reviewer report: Reviewer response for version 2

**DOI:** 10.5256/f1000research.161677.r255630

**Published:** 2024-04-10

**Authors:** Francisco Ponce-Gordo

**Affiliations:** 1 Complutense University of Madrid, Madrid, Spain

General comments

 Please correct the names of parasite species throughout the text. For the amoeba, it should be *Entamoeba* (not Entameobia). While the name *Giardia lamblia* is commonly used in medical literature, it is a synonym of either *Giardia duodenalis, Giardia intestinalis, or Giardia enterica.* Please refer to the taxonomic revision made by Wielinga et al. (2023)[Ref 1]. Even in the GenBank database, the entries identified as *G. lamblia *by different authors are identified in the source organism field as *G. intestinalis.*

 In this study, the methodology (multiplex real-time PCR)

 is based on a previous paper in which the human species were identified as monotypic, as *G. intestinalis (= G. lamblia) and C. parvum*; however, now these species have been separated into *G. duodenalis/G. intestinalis/G. enterica and C. parvum/C. hominis*, and all of which can infect humans; Since precise identification ...". What I want to say is that only *G. intestinalis and C. parvum* were considered in the study where the multiplex PCR was described because at that time these species were the only ones accepted.Most likely, if the study were carried out now, the authors would have said *Giardia spp. *and *Cryptosporidium spp.*, because the methodology they developed does not allow to differentiate between *G. duodenalis-*

*G. intestinalis-G. enterica*, or between *C. parvum-*

*C. hominis*; all these species are now accepted as different but are morphologically indistinguishable.

 When first mentioned, the species name should be indicated in full. As the abstract and the main text are usually considered different texts, please mention the species' full name in the main text when first mentioned, even if the species have been mentioned in the abstract.

 Sometimes, the text is hard to understand. Please revise typographical errors (words, parentheses, spaces, punctuation signs), and grammar errors (e.g., the use of prepositions). A sentence should not start with an abbreviation or with a number in digits; in such cases, the number should be written in text. For example, in the Abstract-results section, it should start: "Four hundred and thirty-seven children with acute diarrhea ...". In the same paragraph, there is a sentence that should not start as "G. lamblia" but as "Giardia spp. (18.8%) and ...".

 Comments on each section:

 In keywords, I suggest including at least the genus names of the parasites: *Giardia, Cryptosporidium, Entamoeba (or Giardia spp., Cryptosporidium spp., Entamoeba histolytica*).

 In the Introduction, it sounds strange to indicate "diarrhea is estimated to have killed 500,667 children". Such a level of precision seems to be not an estimation but a precise count. As there are probably cases that are not reported, I suggest rounding the number to "more than 500,000 children".

 In the methods, please rewrite the paragraph about participants; it is hardly understandable.

 In the results, in the "prevalence of parasitic infections" section, the genetic analysis is supposed to unambiguously diagnose *E. histolytica, Giardia spp., and Cryptosporidium spp*.; then, "A potential protozoal parasite was diagnosed ..." would be incorrect; it would be better to say "Protozoa were diagnosed in 166/437 (35.5%) cases, among which ... . None of them were detected in 282 (64.5%) diarrheal cases".

 In the "Distribution of parasitic infection by age, sex, and seasonality" subsection, please add the number of positive/total samples where appropriate, to keep uniformity and clarify if the percentages were calculated as positive/total cases within each age range, or as positive/total number of cases. For example, if there were 143 positive cases in the <2 yo group, and 12 in the 2-4 yo group, the correct percentages would not be 143/437 (32.7%) and 12/437 (2.7%), but 143/155 (92.2%) and 12/32 (37.5%). Also, add the numbers to tables 2-5.

 In this same subsection, the sentence "Diarrhea remains the leading cause and illness in children <2 years" is not a valid statement, as all samples considered were from symptomatic (diarrheic) patients, but no info is provided about the total number of persons (symptomatic and asymptomatic) in each age group in the population from which this group of patients was investigated.

 In the results, nothing is said about the results of the statistical analysis (apart from the sentence "the comparisons between these variables were not relevant"). It seems that there are clear differences at least between the age groups. There are some comments in discussion (paragraph #4), but the data should be given in results.

 In the discussion, please avoid repetition of data already provided. For example, paragraph #2 says the same thing already mentioned in the introduction and methods sections; paragraph #3 is a repetition of results. In paragraph #5, it is not necessary to repeat the numbers of samples and percentages; they are already given in results. There are texts that can be reduced in extension and/or removed.

 In the paragraph starting "The result revealed a higher prevalence ...", please modify it to something like "... revealed a higher prevalence (35.5%) of protozoa capable of causing diarrhea disease in children under 5 years old".

 In the paragraph before the last one, the sentence "It should be noted that no protozoan pathogen was detected in stool samples, ..." is it supposed to refer to the study (16) carried out in Khartoum state?

 Expanding comments on the review form.

 1. The literature is correct, however the text is sometimes hard to read and confuse. Results should be better presented. The name of the organisms should be revised and corrected.

 2. The results of the statistical analysis should be provided in the text of the results section, or in a table. The correct percentage values should be re-calculated.

 3. There is not a specific conclusions section. About the comments In the last paragraph of discussion, the authors stated:

 - "The present study furnished some crucial insights into the protozoan cause of child diarrhea in Khartoum State". What are those "crucial insights"?

 - "Findings will help us understand the importance of parasitic diarrhea in children younger than five years". What are this importance? (please note that only about one third of the diarrheal samples were positive to protozoa; what about the other two thirds?).

 - "This study calls for the establishment of sensitive and specific molecular techniques". There are no comparative data on parasite diagnosis using non-molecular methods. It would be of great help to support this statement, the availablity of data from microscopical observations in stained/unstained preparations of the same samples, to make comparisons and draw conclusions.

Is the work clearly and accurately presented and does it cite the current literature?

Partly

If applicable, is the statistical analysis and its interpretation appropriate?

Partly

Are all the source data underlying the results available to ensure full reproducibility?

Yes

Is the study design appropriate and is the work technically sound?

Yes

Are the conclusions drawn adequately supported by the results?

Partly

Are sufficient details of methods and analysis provided to allow replication by others?

Yes

Reviewer Expertise:

Parasitology - Protozoology - molecular taxonomy

I confirm that I have read this submission and believe that I have an appropriate level of expertise to confirm that it is of an acceptable scientific standard, however I have significant reservations, as outlined above.

## References

[1] : Proposed taxonomic revision of Giardia duodenalis. *Infect Genet Evol* .2023;111: 10.1016/j.meegid.2023.105430 105430 10.1016/j.meegid.2023.105430 36972861

# Response

**Published:** 2024-06-04

**Authors:** Mosab Adam

**Affiliations:** Central laboratory -Sudan, Sudan

Thanks for the valuable comment, which raised the quality of the article. I revised your comment throughout the article as follows. 

 We made revisions throughout the text with a valuable comment throughout the article with a tracker change, as follows

 - we revised the typographical errors and grammar throughout the article

 - we made the change of name of the pathogen text (giardia lamblia to giardia spp and intestinalis as the reviewer suggested, which was precise; however, for C.parvum, I checked the primer that was used in the study through the BLAST  technique, and we found that it was specific for C.parvum and not detect .C.hominis, 

 -The correct percentage values were re-calculated from a total of positives in the table and changed in all article  section 

 we add a section on statistical analysis  in the result, as also presented  in the tables, and we add a subsection of total cases to each of Tables 2,3,4

 - we revised the discussion by removing repeated sentences, and we removed the points you mention 

 -we add conclusion suction

 -regarding the two-thirds of the samples were negative, I already mentioned in the discussion by proven 

 that infections with other pathogens like viruses and bacteria also may be due to noninfectious reasons like hypersensitivity to certain food ingredients; besides that, we conducted another study to detect other causes of viral and bacteria in Khartoum state in the flowing link (https://pubmed.ncbi.nlm.nih.gov/29487571/)

 All our study samples were collected from symptomatic children with diarrhea admitted to the hospital for 1 to 4 days.

 Unfortunately, we could not investigate using a non-molecular technique; hence, we collected it in Sudan and tested it in China. There, the laboratory has to use only facility-molecular techn

# Reviewer report: Reviewer response for version 1

**DOI:** 10.5256/f1000research.135782.r179577

**Published:** 2023-08-01

**Authors:** Jean Bernard Lekana-Douki

**Affiliations:** 1 University of Health Sciences, Owendo, Gabon; 2 Université Des Sciences de La Santé, Libreville, Gabon

The article titled ‘ **Molecular survey of certain protozoan agents that cause diarrhea in children in Sudan**” by Mosab Adam et *al*. described the identification of three main intestinal protozoan parasite ( *Cyptosprodium parvum*, *Giardia lamblia* and *Entamoeba histolytica*) in Sudan. While the paper has merit, there are a few major challenges with the paper in the current format:

 Page 1 (of the pdf version of the article):

**Abstract**

 Introduction: Changed ‘’aged less than five years” by ‘’children”. Changed “ *Cryptosporidium spp*.” by “ *Cryptosporidium parvum*” and “ *Giardia lambelia*” by “ *Giardia lamblia*”.

 Methods: This section isn’t sufficiently clear and precise. Please make the necessary changes. For example, you might say: “We conducted a cross-sectional survey among children under five years of age hospitalized with acute diarrhea between April to December 2014. Diarrheal stool samples were collected and examined for *E. histolytica*, *C. parvum* and *G. lamblia* using a multiplex real-time PCR method”.

 The number of children included in the study must appear in the results section.

 Results: Please make the necessary changes. For example, you might say: “A total of the 434 acute diarrheal children were included in this study”, following by average mean ± Standard deviation, median age and sex-ratio of this children, before presented global prevalence of parasitic infections, frequency of co-infection and more…

 Discussion? I think the made a mistake in the title section of the abstract. Please write “conclusion” instead of “discussion”.

 Page 3:

**Introduction**

 Sentence 1: The two references cited are old and inappropriate. I recommend citing the WHO online document (Diarrhoeal disease: key facts, May 2, 2017) which is recent and, in my opinion, suitable for this affirmation.

 Sentence 2: You say that diarrhea remains a major problem in terms of morbidity and mortality in children, which is true, but the references cited are not appropriate for this affirmation. I therefore recommend that you replace them with the following articles:

1. Estimates of the global, regional, and national morbidity, mortality, and aetiologies of diarrhoea in 195 countries: a systematic analysis for the Global Burden of Disease Study 2016 (Christopher Troeger et *al*., 2018);
2. Mapping geographical inequalities in childhood diarrhoeal morbidity and mortality in low-income and middle-income countries, 2000-17: analysis for the Global Burden of Disease Study 2017. Lancet. 2020 Jun 6;395(10239):1779-1801. doi: 10.1016/S0140-6736(20)30114-8.
3. GBD 2017 Causes of Death Collaborators. Global, regional, and national age-sex-specific mortality for 282 causes of death in 195 countries and territories, 1980-2017: a systematic analysis for the Global Burden of Disease Study 2017. Lancet. 2018 Nov 10;392(10159):1736-1788. doi: 10.1016/S0140-6736(18)32203-7.

Sentence 3: You claim: “More than 1 billion episodes occur […] than 5 years in developing countries”. This assertion is out of date, because thanks to advances in vaccination (e.g., the extension of rotavirus vaccination) and treatment (e.g., ORS), the burden of diarrheal disease has fallen considerably. Studies show that, over the last three decades, children under 5 years of age have accounted for the majority of deaths from diarrhoeal diseases, with around 1.7 million children in this age group dying in 1990. Your figure of 2.5 million deaths among children under 5 in developing countries is incorrect. Today, interesting data on mortality linked to diarrheal diseases in children are shown by:

1. WHO, title: Diarrheal diseases: highlights, posted online May 2, 2017.

This online document shows that there are around 1.7 billion cases of childhood diarrhea each year worldwide, and that diarrhea kills 525,000 children under the age of 5 each year).

1. Bernadeta Dadonaite, Hannah Ritchie and Max Roser (2018) - "Diarrheal diseases". Published online at OurWorldInData.org. Retrieved from: 'https://ourworldindata.org/diarrheal-diseases' [Online Resource].

According to this article available online, diarrhea killed 500,667 children under 5 worldwide in 2019.

1. Alternatively, you could say: “There are around 1.7 billion cases of childhood diarrhea each year, and diarrhea is estimated to have killed 500,667 children under the age of 5 worldwide in 2019”. (In this case, you'll need to cite the two online articles above).

I recommend that you modify sentence 3 according to one of the three choices I've indicated above.

 Sentence 7: There's an error in the reference numbering; instead of reference number 10, you've written reference number 15. Please correct this error.

 Overall, the introductory section needs to be revised, focusing on the most relevant references related to the topic and **the most recent**, preferably less than 5 years old, as I demonstrated above.

**Methods**

 For greater clarity, please organize the following additional sections:

1. Ethical considerations ;
2. Design, area and period of study
3. Participants (inclusion and exclusion criteria), sample collection and storage
4. Nucleic acid extraction (already present)
5. PCR amplification and parasite detection (already present)
6. Statistical analysis (this is currently missing entirely).

Data on the total number of samples collected, as well as frequencies of hospitalized diarrheic children can be presented in the "results" section (below) in a section entitled "demographic data on the study population".

 Section “PCR amplification and parasite detection”:

 In the last sentence, the authors state that "Table 1 shows the sequences of the primers, probes and targeted genes, as well as the expected sizes", which is partially true as the expected sizes are not shown in Table 1. Please make the necessary corrections.

 Table 1 also contains several references, both for organisms (reference number 5) and probes (reference numbers 16 and 19). I think this is a mistake! If not, please formulate appropriate sentences citing these references in the text before putting them in Table 1.

**Results**

 There is some confusion between *Cryptosporidium* spp and *Cryptosporidium parvum* in this section. *Cryptosporidium* spp. refers to the genus *Cryptosporidium*, which includes over 30 species identified in mammals ( *C. parvum* and *C. hominis* followed by *C. mealegridis* are the main species identified in humans), while *C. parvum* refers to a species of the genus *Cryptoporidium*, which is a so-called zoonotic species, unlike *C. hominis*, which is anthroponotic. I think you mean *C. parvum*, since according to the Verweij et al article first quoted in the methods section, the *Cryptosporidium* primers used were specific to *C. parvum*. Modifications therefore need to be made to this part of the work, in particular to the text and Tables 3, 4 and 5, as well as to the discussion.

 For greater clarity, please organize the following additional sections:

1. Demographic data
2. Prevalence of parasitic infections (modify figure 1 into diagram)
3. Parasitic mono- and co-infections (modify table 5)
4. Distribution of mono- and co-infections by sex, age and seasonality.

**Discussion**

 Paragraph 5: the last quotation (reference number 13) does not correspond to the penultimate sentence (But our result differed [...] from the age of the group by ≤ 2 years;).

 Overall, the research results are not sufficiently valued. Limitations or weaknesses are not sufficiently highlighted.

**Is the work clearly and accurately presented and does it cite the current literature?**

 No. Some of the bibliographical references cited are not appropriate to the assertion or argument put forward. In addition, there is an error in the numbering of the references cited by the authors.

**Is the study design appropriate and is the work technically sound?**

 Partly. Although the work may have academic value, the study design is a little confusing, as some of the results on the demographic characteristics of the children included in the study are mixed with information on the study design. Inclusion and exclusion criteria for hospitalized children are not clearly presented. The study design needs to be properly reformulated.

**Are sufficient details of methods and analysis provided to allow replication by others?**

 Partly.

**If applicable, is the statistical analysis and its interpretation appropriate?**

 No. The statistical analyses carried out have not been set out in the "Methods" section, making interpretation of the results inappropriate. Furthermore, when percentages are mentioned, it is preferable to provide the figures used to calculate the prevalence or percentage, enabling the reader to interpret and evaluate the results more clearly.

**Are all the source data underlying the results available to ensure full reproducibility?**

 Partly.

**Are the conclusions drawn adequately supported by the results?**

 Partly.

Is the work clearly and accurately presented and does it cite the current literature?

No

If applicable, is the statistical analysis and its interpretation appropriate?

Partly

Are all the source data underlying the results available to ensure full reproducibility?

Partly

Is the study design appropriate and is the work technically sound?

Partly

Are the conclusions drawn adequately supported by the results?

Partly

Are sufficient details of methods and analysis provided to allow replication by others?

Partly

Reviewer Expertise:

Epidemiology of Infectious diseases

I confirm that I have read this submission and believe that I have an appropriate level of expertise to confirm that it is of an acceptable scientific standard, however I have significant reservations, as outlined above.

## References

[1] : Diarrhoeal disease: key facts. *May 2, 2017* . Reference source

[2] : Estimates of the global, regional, and national morbidity, mortality, and aetiologies of diarrhoea in 195 countries: a systematic analysis for the Global Burden of Disease Study 2016. *The Lancet Infectious Diseases* .2018;18(11) : 10.1016/S1473-3099(18)30362-1 1211-1228 10.1016/S1473-3099(18)30362-1 30243583 PMC6202444

[3] : Mapping geographical inequalities in childhood diarrhoeal morbidity and mortality in low-income and middle-income countries, 2000–17: analysis for the Global Burden of Disease Study 2017. *The Lancet* .2020;395(10239) : 10.1016/S0140-6736(20)30114-8 1779-1801 10.1016/S0140-6736(20)30114-8 PMC731459932513411

[4] : Global, regional, and national age-sex-specific mortality for 282 causes of death in 195 countries and territories, 1980–2017: a systematic analysis for the Global Burden of Disease Study 2017. *The Lancet* .2018;392(10159) : 10.1016/S0140-6736(18)32203-7 1736-1788 10.1016/S0140-6736(18)32203-7 PMC622760630496103

[5] : "Diarrheal Diseases". *Published online at OurWorldInData.org* .2023; Reference source

# Response

**Published:** 2024-01-27

**Authors:** Mosab Adam

**Affiliations:** Central laboratory -Sudan, Sudan

Thanks for these informative comments. I found it precise and increased the quality of our manuscript. 

 We took all reviewer comments, made corrections throughout the paper, and updated this version.

 1. rewrote the abstract section and updated it according to the reviewer's suggesting 

 2. we update the introduction section by rewriting the updated information, adding new updated references, and deleting the old ones

 3. rewrote the method section, added the missing information, and updated carefully with the help of reviewers' comments 

 4. rewrote the result section, updated and classified it as the subtitle, added the statistical part, updated the tables and the diagram 

 5. update the discussion section and address the reviewer's comments

# Response

**Published:** 2024-06-04

**Authors:** Mosab Adam

**Affiliations:** Central laboratory -Sudan, Sudan

Thanks for your valuable comments 

 I corrected that point you mentioned as I changed gender to sex, then rewrote the sentence  in a clear way and corrected the sentence in the text that you commented on it

# Reviewer report: Reviewer response for version 1

**DOI:** 10.5256/f1000research.135782.r179572

**Published:** 2023-07-26

**Authors:** Monira Sarmin

**Affiliations:** 1 International Centre for Diarrhoeal Disease Research, Dhaka, Bangladesh

This is an important study,

 Write up needs a lot of improvement. Introduction is well written. However other sections need care especially the discussion, results were repeated many a times.

 I am adding my observations:

**Methods: **

1. Duration of the study?
2. How the sample size 437 were fixed? Did you calculate the sample size?
3. What was the sampling methods?
4. Did you enroll all children? Please add a study flow diagram.
5. What were the eligibility criteria?
6. Description of the study site might help the reader to gasp the scenario.
7. Is Jan- March winter season? Why excluded?
8. Methods section also need a description of the statistical analysis. Which analytical tools were used for data analysis?
9. Needs discussion with a statistician.

**Results:**

1. Usually a pie chart consists of 100%, though there is a label of 18.8, visually it is >50%, So, the figure 1 needs to corrected. You can build a pie of pie or bar of pie instead.
2. Table 4 indicates season as Autumn and summer, there is no elaboration in the method. Rather, it was labelled as dry and rainy seasons. Do they differ?
3. Did you collect only data on age, sex, season?
4. Their nutritional status, socio-economic condition, vaccination, housing, water supply Presence/absence of comorbidities, other laboratory analysis, outcome of this admission also needs to be incorporated.
5. Patient data were collected through a structured questionnaire, including age, gender, and season. This statement needs modification. We collect data on biological sex, not on gender. There is difference between sex and gender.
6. As much more samples were collected from boys (276) than from girls (161) in the age group ≤2 years (403, 92.2%), the comparisons between these variables were not relevant.—This statement is not correct, proportion is the most important indicator. And in the result section, we should only present the results. We have discussion section to accept or refute the results.
7. Did your facility receive more male child, or you have included more male children? What is the national proportion?
8. Table 2,3,4 could be merged to a single table- column containing the name of parasite and rows containing the distribution of age, sex, season and etc.

**Discussion:**

1. The highest rate of parasitic infection was seen in the ≤2 years group (32.7%) and much lower in the >2–≤4 years old group (2.7%) (Table 2). In contrast, the protozoal parasite was not detected in the age group of >4–<5. Why it happened? What is your explanation?
2. This study was conducted during two seasons (autumn and summer), from April to December 2014, however, in methods, July was not included.
3. male-to-female ratio of 1:1.7, this result first appeared in the discussion section, but it should be come in the result section first.
4. If maternal immunity is an important factor against parasitic infection, then it is assumed that older children had more parasitic infection than younger children, but this study did not find that.
5. A limitation section should be added.

Is the work clearly and accurately presented and does it cite the current literature?

Partly

If applicable, is the statistical analysis and its interpretation appropriate?

No

Are all the source data underlying the results available to ensure full reproducibility?

Partly

Is the study design appropriate and is the work technically sound?

Yes

Are the conclusions drawn adequately supported by the results?

Partly

Are sufficient details of methods and analysis provided to allow replication by others?

Partly

Reviewer Expertise:

NA

I confirm that I have read this submission and believe that I have an appropriate level of expertise to confirm that it is of an acceptable scientific standard, however I have significant reservations, as outlined above.

# Response

**Published:** 2024-01-27

**Authors:** Mosab Adam

**Affiliations:** Central laboratory -Sudan, Sudan

Thanks for these informative comments ts really raise up our manuscript 

 We took all reviewer comments, made corrections throughout the paper, and updated this version.

 1. rewrote the abstract section and updated it according to the reviewer's suggesting 

 2. we update the introduction section by rewriting the updated information, adding new updated references, and deleting the old ones

 3. rewrote the method section, added the missing information, and updated carefully with the help of reviewers' comments 

 4. rewrote the result section, updated and classified it as the subtitle, added the statistical part, updated the tables and the diagram 

 5. update the discussion section and address the reviewer's comments

# Reviewer report: Reviewer response for version 1

**DOI:** 10.5256/f1000research.135782.r160656

**Published:** 2023-02-15

**Authors:** Luther A Bartelt, Renay Ngobeni

**Affiliations:** 1 Division of Infectious Diseases, University of North Carolina at Chapel Hill, Chapel Hill, NC, USA; 2 University of North Carolina at Chapel Hill, Chapel Hill, NC, USA

The investigators report on the findings of 3 protozoans in children under 5 that were hospitalized for acute diarrhea. It is always helpful to have surveillance data for difficult to detect pathogens using molecular methods from a wide range of global sites. The report is therefore helpful in contributing data to improved understanding of local patterns of these globally distributed parasites. 

 The limitations, which the authors need to better explain, include a limited number of demographics variables (resulting in an inability to control for possible confounders that lead to a finding of a difference between M vs F Giardia status for example), the lack of reporting of statistical methods, the lack of reporting of PCR validation steps and whether any biomass or molecular method was used to standardize the DNA extraction, and the lack of a comparator of conventional-based diagnostics that would greatly enhance the differential sensitivity of the molecular methods (as it is one cannot discern if the difference in Cryptosporidium spp. reported in this study compared with others in Sudan as raised in the discussion is due to difference in diagnostic tools or difference in study population).

 Also, the title is misleading. The study population appears to be children who were hospitalized for acute diarrhea. This population is different from a population consisting of diarrhea of any severity. We recommend the title be modified to better reflect the population studied: **Molecular prevalence of three intestinal protozoa in young children hospitalized for acute diarrhea in rural Sudan.** If this is a population of children with moderate-to-severe diarrhea (was there any tool to assess diarrhea severity other than hospitalization?), then the discussion should address and cite other studies specific to this acutely ill population. 

**General**:

 Spelling and grammar need to be reviewed throughout. For example, "lambelia" and Entameobia are wrong. For example, in the results section, should 'the comparisons between these variables were not relevant' read 'the comparisons...were not statistically significant'? It is not accurate to claim a comparison is irrelevant. Please clarify, or change all references to specific Cryptosporidium species to Cryptosporidium spp. (or show specific sequencing results that were used to determine the specific species). 

**Abstract:**

 Please provide consistent reporting in the results section. The N (%) notation as in the first sentence should be reported for all. 

**Methods**:

 Please include validation methods for the triple-plex PCR (eg. were there positive controls)? Is there conventional diagnostic data to accompany the PCR results? How did the team decide to dilute all fecal specimens into 10% saline? Was anything done to normalize the amount of fecal material at baseline (some diarrheal samples may already me more 'dilute' than others). QIAmp DNA stool extraction kits may be suboptimal for DNA extraction. Were any steps taken to enrich DNA from parasite (oo)cysts (for example, freeze-thaw and/or bead beating)?

 For improved clarity, please organize with the following additional sections: 

1. Ethical consideration
2. Study area and participants
3. Sample collection and storage
4. Nucleic acid extraction ( *already present)*
5. PCR amplification and parasite detection ( *already present*). 
6. **Statistical analysis (this is currently missing entirely)**

**Results:**

 Please provide a table with the demographics data for all variables recorded from the questionnaire. ****As in the abstract, please include N (%) notations for all reported results for consistency. Please apply consistent use of a) gender-specific terms (male and female rather than flipping to 'boys and girls'), b) seasonality (hot-dry vs rainy rather than flipping to summer vs autumn in the discussion). The PCR reported does not appear to be specific for Cryptosporidium parvum (as claimed in the abstract). Please clarify what "1" and "2" mean in the source data and why some columns are incomplete and why some text is red and other text is black.

**Discussion:**

 Please expand on the importance of the finding of M vs F differences in G lamblia positivity, and in context with other published literature. Only one citation is used here, and little is articulated about the statement of "Kwam H." Important questions here would relate to exposure risks versus some other reason why male and female children would have different susceptibilities to infection. Please clarify this statement: "due to noninfectious reasons like hypersensitivity to certain food ingredients and weaning diarrhea that result of the inability of an underdeveloped child intestine to metabolize the food". What are the clinical/treatment implications of finding 35% prevalence of these 3 protozoa in children hospitalized in rural Sudan?

Is the work clearly and accurately presented and does it cite the current literature?

Partly

If applicable, is the statistical analysis and its interpretation appropriate?

No

Are all the source data underlying the results available to ensure full reproducibility?

Partly

Is the study design appropriate and is the work technically sound?

Partly

Are the conclusions drawn adequately supported by the results?

Partly

Are sufficient details of methods and analysis provided to allow replication by others?

Partly

Reviewer Expertise:

Infectious diseases, intestinal protozoa epidemiology and pathogenesis, malnutrition, microbiota

We confirm that we have read this submission and believe that we have an appropriate level of expertise to state that we do not consider it to be of an acceptable scientific standard, for reasons outlined above.
